# Doxorubicin-induced necrosis is mediated by poly-(ADP-ribose) polymerase 1 (PARP1) but is independent of p53

**DOI:** 10.1038/srep15798

**Published:** 2015-11-02

**Authors:** Hyeon-Jun Shin, Hyuk-Kwon Kwon, Jae-Hyeok Lee, Xiangai Gui, Asma Achek, Jae-Ho Kim, Sangdun Choi

**Affiliations:** 1 Department of Molecular Science and Technology, Ajou University, Suwon 443-749, Korea

## Abstract

Necrosis, unregulated cell death, is characterized by plasma membrane rupture as well as nuclear and cellular swelling. However, it has recently been reported that necrosis is a regulated form of cell death mediated by poly-(ADP-ribose) polymerase 1 (PARP1). PARP1 is thought to mediate necrosis by inducing DNA damage, although this remains unconfirmed. In this study, we examined the mechanisms of PARP1-mediated necrosis following doxorubicin (DOX)-induced DNA damage in human kidney proximal tubular (HK-2) cells. DOX initiated DNA damage response (DDR) and upregulated PARP1 and p53 expression, resulting in morphological changes similar to those observed during necrosis. Additionally, DOX induced mitochondrial hyper-activation, as evidenced by increased mitochondrial respiration and cytosolic ATP (cATP) production. However, DOX affected mitochondrial mass. DOX-induced DNA damage, cytosolic reactive oxygen species (cROS) generation, and mitochondrial hyper-activation decreased in cells with inhibited PARP1 expression, while generation of nitric oxide (NO) and mitochondrial ROS (mROS) remained unaffected. Moreover, DOX-induced DNA damage, cell cycle changes, and oxidative stress were not affected by p53 inhibition. These findings suggest that DNA damage induced necrosis through a PARP1-dependent and p53-independent pathway.

Traditionally, cell death processes have been classified as apoptosis or necrosis. Apoptosis is the process of regulated cell death, while necrosis refers to unregulated cell death triggered by chemotherapeutic drugs or other insults^1^. Morphologically, the two processes differ in that apoptosis involves cell shrinkage, pyknosis, and the generation of apoptotic bodies, while necrotic cells undergo plasma membrane rupture and nuclear and cellular swelling^2^. Chan *et al.* reported that tumor necrosis factor (TNF) or TNF-related apoptosis-inducing ligand (TRAIL) induce necrosis via the receptor-interacting protein (RIP) by inhibiting caspase 8^3^,^4^. The formation of the necrosome by RIP homotypic interaction motif (RHIM) domains of RIP1 and RIP3, recruits mixed lineage kinase domain-like (MLKL) protein, which activates TNF-induced necrosis^5^,^6^. TNF induces necrotic cell death through RIP-mediated reactive oxygen species (ROS) generation when caspase activity is inhibited^7^. Moreover, TNF-induced ROS generation, via NADPH oxidase 1 (NOX1), in the plasma membrane has been reported to contribute to necrotic cell death^8^. In contrast, another study showed that TNF-induced necrotic cell death was independent of ROS generation in human colon adenocarcinoma (HT-29) cells^9^. Moreover, it has been reported that, in addition to TNF-receptors, the activation of Toll-like receptors (TLRs) by pathogen-associated molecular patterns (PAMPs) or damage-associated molecular patterns (DAMPs) might lead to necrosis^10^,^11^. The interaction of TLR4 with a component of the outer membrane of gram-negative bacteria, lipopolysaccharide (LPS), causes necrosis and inhibits caspase 8 activation in macrophage cells^12^. Furthermore, the activation of TLR3 by polyinosinic:polycytidylic acid [poly(I:C)] and of TLR4 by LPS was reported to induce necrosis through RIP3-mediated ROS generation in caspase-inhibited macrophage cells^13^. Taken together, these findings showed that different pathways are associated with necrosis, resulting in the onset of various diseases, such as cardiovascular disease, Alzheimer’s disease, and cancer^14^,^15^. Moreover, these results also suggested that necrosis is a form of regulated cell death (also called programmed necrosis or necroptosis), the molecular mechanisms of which are not yet fully understood.

Poly-(ADP-ribose) polymerase 1 (PARP1) is an important nuclear protein comprising a DNA-binding domain containing zinc fingers in the N-terminal domain, an automodification domain in the central region, and a catalytic domain in the C-terminal domain. The zinc fingers of the DNA-binding domain recognize DNA breaks, and result in sequential poly-(ADP-ribosyl)ation using nicotinamide adenine dinucleotide (NAD^+^) and adenosine triphosphate (ATP) via the catalytic domain. This process is involved in DDR signaling pathways, such as DNA damage repair and cell death^16^. Additionally, the activation of PARP1 mediates a range of functions, including oxidative stress, mitochondrial dynamics, inflammatory responses, and cell death signaling pathways in both normal and cancer cells^17^,^18^. However, hyper-activation of PARP1 enhances apoptosis-inducing factor (AIF) production, which after its release from the mitochondria translocates to the nucleus, ultimately triggering DNA fragmentation, NAD^+^ and ATP depletion, and necrosis. The previously described process is known as parthanatos (PARP1-dependent cell death)^19^,^20^,^21^. TRAIL-induced necroptosis is mediated by RIP1/3-dependent PARP1 activation in various cell lines^22^. Additionally, hyper-activation of PARP1 promotes the expression of pro-inflammatory genes, which can aggravate various cardiovascular diseases, such as myocardial infarction and coronary artery disease^23^. Polymorphisms in *PARP1* are also closely related to the development of Alzheimer’s and Parkinson’s diseases^24^,^25^. In particular, recent studies have shown that cisplatin, a DNA damage-inducing platinum-based drug, increases the expression of PARP1 during kidney injury. PARP-initiated ATP depletion as well as generation of oxidative stress products causes nephrotoxicity by enhancing necrosis^26^. Cisplatin enhances necrotic cell death through the activation of PARP1 in human (HK-2), mouse (MCT), and pig (LLC-PK1) kidney proximal tubular cells^27^. Although the molecular mechanisms of necrosis or necroptosis are currently being studied actively, the potential roles of PARP1 in mitochondria-, oxidative stress-, and ATP-related pathways during DNA damage-induced necrosis are not yet fully understood.

In this study, we investigated the mechanisms through which PARP1 mediates doxorubicin (DOX)-induced necrosis by examining mitochondrial dynamics and ROS generation in HK-2 cells. Additionally, we examined the morphological changes that occur during necrotic cell death by using carbon nanotube (CNT) atomic-force microscopy (AFM) probes.

## Results

### DOX induces DNA damage, cell cycle arrest, and the expression of PARP1 and p53

**Figure 1:**
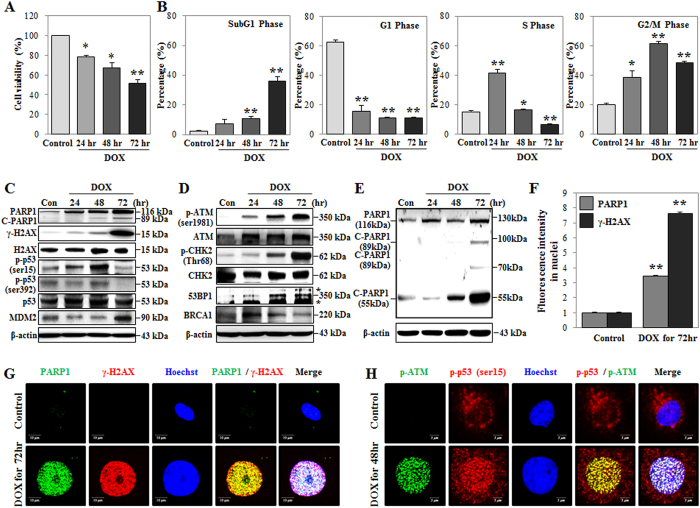
Doxorubicin (DOX) induces DNA damage and G_2_/M- to S-phase arrest. HK-2 cells were treated with DOX (1 μM) for the indicated times. (**A**) Cell viability was measured using MTT assays and detected by a microplate reader. (**B**) DNA content and cell cycle were analyzed using PI staining and detected by FACS analysis. The percentage of cells in each phase is presented in the histogram. (**C**−**E**) Expression of PARP1 (116 kDa), cleaved-PARP1 (C-PARP1; 89 kDa and 55 kDa), γ-H2AX^ser139^ (15 kDa), H2AX (15 kDa), MDM2 (90 kDa), 53BP1 (350 kDa), BRCA1 (350 kDa), p-p53^ser15^ (53-kDa), p-p53^ser392^ (53 kDa), p53 (53 kDa), p-ATM^ser1981^ (350 kDa), ATM (350 kDa), p-CHK2^Thr68^ (62 kDa), and CHK2 (62 kDa) proteins were measured using western blot analysis of whole-cell extracts. β-Actin was used as a loading control (*nonspecific band). The gels were performed under the same experimental conditions. (**F**) Nuclear expression of γ-H2AX^ser139^ and PARP1 was measured using immunofluorescence staining and a Cellomics ArrayScan HCS Reader analysis at 72 h. At least 200 cells were analyzed per sample. (**G**) Expression levels of γ-H2AX^ser139^/PARP1 at 72 h and p-p53^ser15^/p-ATM^ser1981^ at 48 h were measured using immunofluorescence staining and confocal microscopy analysis. Nuclei were stained with Hoechst stain (blue color). Scale bar: 10 μm. See Supplementary Information Figure S1A and B. All histograms shown are the average of the results of three independent experiments (*P < 0.05, **P < 0.01 compared to the control).

In mammalian cells, DOX induced DNA damage through the topoisomerase II (TOP II) complex, which is related to DDR signaling pathways involving a DNA-damage sensor, mediator, and effector proteins, including PARP1, H2A histone family member X (H2AX), ataxia telangiectasia mutated (ATM), checkpoint kinase 2 (CHK2), p53, p53-binding protein 1 (53BP1), and breast cancer 1 early onset (BRCA1)^28^,^29^,^30^. Therefore, we used HK-2 cells to examine whether DOX regulates cell viability, DNA damage, cell cycle arrest, and DDR protein expression levels. DOX increased the percentages of cells in the subG1 phase (indicating DNA damage), and in the S and G2/M phases; further, we observed that DOX decreased cell viability (Fig. 1A,B). Moreover, western blot analysis showed that DOX time-dependently induced the expression of PARP1 and the phosphorylation of H2AX (γ-H2AX) (Fig. 1C,E). DOX significantly increased the expression of PARP1 and γ-H2AX in the nucleus, as evidenced by increased fluorescence intensity observed in confocal microscopy and Cellomics ArrayScan HCS Reader analysis (Fig. 1F,G and Supplementary Information Figure S1A). The morphological changes characteristic of cells undergoing necrotic cell death include swelling of both the nucleus and the cell^31^. The nuclear area of DOX-treated cells increased significantly compared to the nuclear area of control cells (Fig. 1G,H) with quantitative increases measured using a Cellomics ArrayScan HCS Reader in at least 200 cells (Supplementary Information Figure S1D and E). A previous study showed that cleavage of PARP1 occurs during both necrosis and apoptosis. In the case of necrosis, PARP1 was cleaved by lysosomal protease, resulting in a 55-kDa cleaved-PARP1 (C-PARP1) protein, whereas in the case of apoptosis, caspase 3 cleaves PARP1 to produce an 89-kDa C-PARP1 protein^32^. In this study, we observed that DOX treatment significantly increased the expression of PARP1 and C-PARP1 (55-kDa) in a time-dependent manner. In contrast, the level of C-PARP1 (89-kDa) increased only after 72 h of DOX treatment. The expression level of C-PARP1 (89-kDa) was also lower than that of the C-PARP1 (55-kDa) cleavage product (Fig. 1E). Moreover, the levels of DNA damage mediators and effectors, such as phospho-ATM (p-ATM), phospho-CHK2 (p-CHK2), and 53BP1 increased, while that of BRCA1 decreased in response to DOX treatment (Fig. 1D). The phosphorylation of p53 at serine 15 (p-p53^ser15^) and serine 392 (p-p53^ser392^) increased, and the expression of murine double-minute 2 (MDM2, a negative regulator of p53) decreased in response to a 48-h DOX treatment and returned to basal levels at later time points (Fig. 1C). The fluorescence intensities of p-p53^ser15^ and p-ATM and the extent of their co-localization in the nucleus increased at 48 h, while p-p53^ser392^ was only observed in the cytosol (Fig. 1H and Supplementary Information Figure S1B and C). These results demonstrated the DOX-induced expression of PARP1 and p53, DNA damage, cell cycle arrest, and nuclear swelling.

### DOX and ETO differently regulate mitochondrial dynamics, cATP production, cell swelling, and cell death

**Figure 2:**
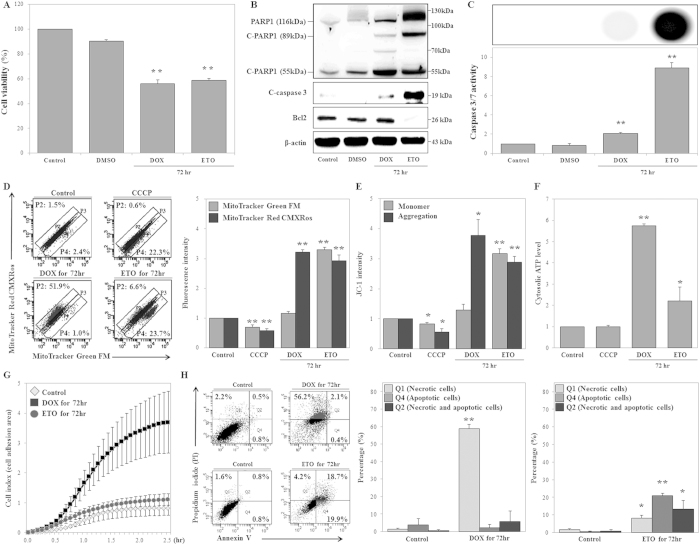
Doxorubicin (DOX) and etoposide (ETO) differentially regulate mitochondrial mass via respiration, cell adhesion, and cell death. (**A**–**C**) HK-2 cells were treated with DOX (1 μM) or ETO (50 μM) for 72 h. (A) Cell viability was measured using MTT assays, and signals were detected using a microplate reader. (**B**) Expression of PARP1 (116 kDa), cleaved-PARP1 (C-PARP1; 89 kDa and 55 kDa), cleaved-caspase 3 (19 kDa), and BCL2 (26 kDa) were determined using western blot analysis of whole cell extracts. β-Actin was used as a loading control. (**C**) HK-2 and treated cells were counted using a hemocytometer, and same number of cells seeded on plates. Caspase 3/7 activity was measured using Caspase-Glo 3/7 Assays, and signals were detected using a microplate reader, which measured luminescence intensity. (**D–J**) HK-2 cells were treated with DOX (1 μM) and ETO (50 μM) for 72 h, followed by treatment with CCCP (50 μM) for 5 min. (**D**) Mitochondrial mass and respiration were measured using MitoTracker Green FM and Red CMXRos double staining, respectively. Detection was facilitated by FACS analysis, and the percentage of fluorescence-positive cells was measured using dot plot analysis. The histogram shows fluorescence intensities. (**E**) Mitochondrial mass (monomer) and outer membrane potential (ΔΨm; aggregation) were measured using JC-1 staining and detection by FACS analysis. Fluorescence intensities are presented in the histogram. (**F**) HK-2 cells and treated cells were counted using a hemocytometer, and same number of cells was seeded on plates. Cytosolic ATP levels were measured using ATP-based CellTiter-Glo Luminescent Cell Viability Assays and were detected using a microplate reader. (**G**) HK-2 and treated cells were counted using a hemocytometer, and the same number of cells was seeded on a device that allows measurement of the cell-adhesion area by real-time monitoring of impedance, using an xCELLigence System, during the 2.5-h study period. (**H**) Analysis of DOX- and ETO-dependent cell death, including necrosis (Q1), apoptosis (Q4), and necrosis with apoptosis (Q2), as measured using Annexin V and PI double-staining. Necrotic and/or apoptotic cells were detected by FACS analysis. The histogram shows the percentage of cells, and represents the average of the results of three independent experiments (*P < 0.05, **P < 0.01 compared to the control).

The TOP II-targeting drug etoposide (ETO) was shown to induce mitochondrial biogenesis, as evidenced by increased mitochondrial mass and respiration, upon staining with mitochondrial-targeting dyes^33^. Furthermore, chemotherapeutic drugs, such as DOX and mitoxantrone, increase mitochondrial mass during apoptosis in cardiac cells^34^. In our study, ETO (50 μM) reduced cell viability (analogous to DOX) and markedly increased C-PARP1 (89-kDa) levels, Bcl2 degradation, cleaved-caspase 3 expression levels, and caspase 3/7 activity, while the effects of DOX on these parameters were not significant (Fig. 2A–C). Previous studies reported that mitochondrial outer-membrane permeabilization (MOMP) induced the release of numerous cell death factors, such as cytochrome c, endonuclease G, and AIF. In particular, AIF translocates into the nucleus and contributes to cell death^35^,^36^. Thus, we measured the translocation of AIF into the nucleus using western blot analysis at 72 h after DOX and ETO treatment. DOX had no effect on the translocation of cleaved-AIF (57 kDa, the mature form of AIF) into the nucleus, but ETO significantly increased the translocation of cleaved-AIF into the nucleus. (Supplementary Information Figure S2). Moreover, DOX and ETO treatments increased cell size, as evidenced by light scattering experiments and hemocytometer analysis (data not shown). Therefore, we examined whether DOX and ETO induced cell swelling using the xCELLigence system, which uses a gold electrode and real-time monitoring of impedance in order to measure cell proliferation and viability^37^. We modified this system to ensure accurate measurement of cell swelling. Treated cells were harvested, seeded onto the device at the same density, and subjected to analysis of real-time impedance; cells that appeared swelled had increased adhesion areas, thereby increasing impedance. From our xCELLigence results, we found that DOX treatment increased cell swelling (4.5-fold) compared to control during the 2.5-h treatment period, but ETO had no significant effect on cell adhesion area (Fig. 2G). Additionally, DOX significantly increased the number of necrotic cells compared to the control, while the number of apoptotic cells and the number of necrotic with apoptotic cells remained unaffected upon Annexin V/propidium iodide (PI) staining. In contrast, ETO concurrently increased the number of necrotic and apoptotic cells (Fig. 2H). Hence, DOX completely induced necrotic cell death, but ETO induced necrotic and apoptotic cell death in HK-2 cells.

We then investigated whether DOX and ETO regulated mitochondrial dynamics, including mitochondrial mass, respiration, and outer membrane potential (ΔΨm), by staining with MitoTracker Green FM (MTG), MitoTracker Red CMXRos (MTR), and JC-1 dyes. The amount of MTR dye that entered cells depended on the ΔΨm, which itself was a function of the mitochondrial respiration rate. MTG can be used to measure mitochondrial mass, since it enters cells independently of the ΔΨm^38^. JC-1 can be used to calculate ΔΨm from the ratio of the fluorescence intensities of the dye present in the monomeric form to the intensities of the dye in aggregates^39^. We found that carbonyl cyanide 3-chlorophenylhydrazone (CCCP), a known disrupter of ΔΨm, decreased mitochondrial respiration, mass, and ΔΨm compared to the control, confirming the suitability of the experiments (Fig. 2D,E). According to the results obtained using double staining with MTG and MTR, DOX induced accumulation of cells in the P2 region and increased mitochondrial respiration, although no obvious changes in mitochondrial mass were observed. In contrast, ETO induced accumulation of cells in the P4 region and simultaneously increased mitochondrial mass and respiration (Fig. 2D). DOX increased mitochondrial respiration, not mitochondrial mass, while ETO concurrently increased mitochondrial mass and respiration, compared to the control (Fig. 2D and Supplementary Information Figure S3). Similarly, JC-1 staining revealed that both DOX and ETO induced significant increases in ΔΨm (JC-1 aggregation) and confirmed that ETO affected mitochondrial mass (JC-1 monomer), while DOX did not (Fig. 2E). Additionally, DOX- and ETO-treated cells were harvested and seeded on plates at the same density for normalization of cell numbers. These cells were used for cATP levels measurement using ATP-based CellTiter-Glo Luminescent Cell Viability Assay. The cATP levels in both DOX- and ETO-treated cells increased compared to control. Moreover, cATP levels in DOX-treated cells were higher than in ETO-treated cells (Fig. 2F). These findings led us to conclude that mitochondrial hyper-activation (without an effect on mitochondrial mass) and cATP production were critical for DOX-induced necrotic cell death.

### DOX induces DNA damage, cell cycle arrest, and oxidative stress independently of p53

**Figure 3:**
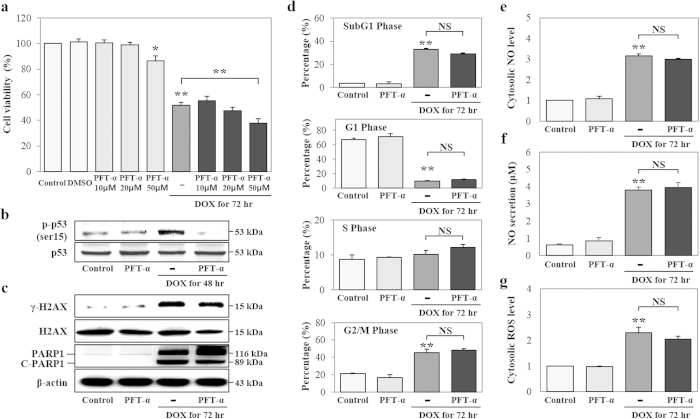
p53 does not affect doxorubicin (DOX)-induced DNA damage, cell cycle arrest, or oxidative stress. HK-2 cells were treated with a pharmacological p53 inhibitor (PFT-α: 10, 20, and 50 μM), DOX (1 μM), or both PFT-α and DOX for 72 h. (**A**) Cell viability was measured using MTT assays and detection with a microplate reader. (**B–G**) We selected PFT-α (20 μM) for the subsequent experiments. (**B**,**C**) Expression of p-p53^ser15^ (53 kDa) and p53 (53 kDa) at 48 h, and γ-H2AX^ser139^ (15 kDa), H2AX (15 kDa), PARP1 (116 kDa), and C-PARP1 (89 kDa) at 72 h were measured by western blot analysis in whole cell extracts. β-Actin was used as a loading control. The gels were run under the same experimental conditions. (**D**) DNA content and cell cycle were assessed by PI staining and subsequent FACS analysis. Data are presented as a percentage of each phase, in histograms. (**E,F**) Cytosolic NO and secreted NO levels were measured by DAF-FM staining and an NO detection kit, using FACS analysis and a microplate reader, respectively. (**G**) Cytosolic ROS levels were measured using DAF-DA staining and detection by FACS analysis. All histograms shown are the average of the results of three independent experiments (*P < 0.05, **P < 0.01, and NS: not statistically significant compared to the control or DOX).

Previous studies showed that the transcription factor p53 binds to DNA promoter sequences, leading to the expression of p53 target genes and thereby regulating cellular processes, such as cell cycle arrest, oxidative stress response, and cell death^40^,^41^. Furthermore, DOX increases p53 activity in normal and tumor cells, and p53 independently advances DOX-induced apoptosis in normal cells, but this process is p53-dependent in tumor cells^42^. Our results showed that DOX increased the phosphorylation and nuclear translocation of p53 (Fig. 1C,H). Thus, we investigated whether p53 was involved in mediating DOX-induced DNA damage, cell cycle arrest, and oxidative stress by using the pharmacological p53 inhibitor pifithrin-α (PFT-α) at 72 h after DOX stimulation. We specifically examined the dose-dependent effects of PFT-α on cell viability. The inhibition of p53 did not have a significant effect on DOX-induced attenuation of cell viability, while 50 μM PFT-α induced significant cytotoxicity; we therefore selected 20 μM PFT-α for the subsequent experiments (Fig. 3A). PFT-α inhibited DOX-induced p-p53^ser15^ expression (Fig. 3B). On the other hand, the inhibition of p53 did not show any effect on DOX-induced expression of γ-H2AX, PARP1, C-PARP1 (89-kDa), or cell cycle arrest, including the SubG1, S, and G2/M phases (Fig. 3C,D). Furthermore, the inhibition of p53 did not affect DOX-induced cytosolic nitric oxide (cNO), secreted NO (sNO), or cROS production (Fig. 3E–G). Therefore, our results showed that DOX induces p53-independent DNA damage, cell cycle arrest, and oxidative stress.

### PARP1 induces mitochondrial hyper-activation, cATP production, cROS generation, and necrosis by DOX, but does not affect mROS and NO generation

**Figure 4:**
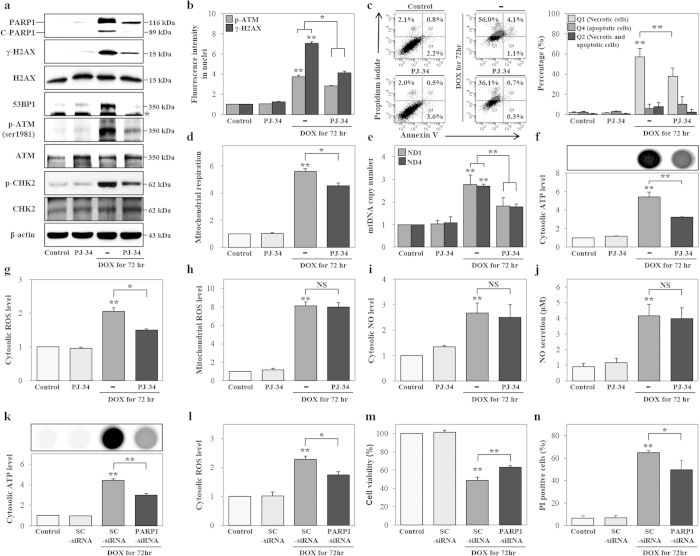
Inhibition of PARP1 suppresses doxorubicin (DOX)-induced DNA damage, mitochondrial hyper-activation, and necrosis. (**A–J**) HK-2 cells were treated with a pharmacological PARP1 inhibitor (PJ-34, 10 μM), DOX (1 μM), or both PJ-34 and DOX for 72 h. (A) Expressions of PARP1 (116 kDa), C-PARP1 (89 kDa), γ-H2AX^ser139^ (15 kDa), H2AX (15 kDa), 53BP1 (350 kDa), p-ATM^ser1981^ (350 kDa), ATM (350 kDa), p-CHK2^Thr68^ (62 kDa), and CHK2 (62 kDa) proteins were measured by western blot in whole cell extracts. (**B**) Levels of p-ATM^ser1981^ and γ-H2AX^ser139^ in the nucleus were measured using immunofluorescence staining and Cellomics ArrayScan HCS Reader. (**C**) Analysis of cell death, including necrosis (Q1), apoptosis (Q4), and necrosis with apoptosis (Q2), as measured using Annexin V and PI double staining. Necrotic and/or apoptotic cells were detected by FACS. (**D**) Mitochondrial respiration levels were measured using MitoTracker Red CMXRos and signals were detected using FACS. (**E**) Genomic DNA was analyzed for mtDNA copy numbers using *ND1-*, *ND4-*, and *GAPDH-*specific primers and real-time PCR. *ND1* and *ND4* copy numbers were normalized to *GAPDH* copy numbers. (**F**) The same number of cells was seeded on plates. Cytosolic ATP levels were measured using ATP-based CellTiter-Glo Luminescent Cell Viability Assays. (**G**,**H**) Cytosolic ROS and mitochondrial ROS levels were measured using DCF-DA and MitoSOX, respectively, and signals were detected by FACS. (I and J) Cytosolic NO and secreted NO levels were measured using DAF-FM and an NO detection kit. (**K**,**N**) HK-2 cells were transfected with SC-siRNA (5 nM) or *PARP1*-siRNA (5 nM) and treated with DOX for 72 h. (**K**) The same number of cells was seeded on plates. Cytosolic ATP levels were measured using ATP-based CellTiter-Glo Luminescent Cell Viability Assay. (**L**) Cytosolic ROS levels were measured using DCF-DA and were detected using FACS. (**M**) Cell viability was measured using MTT assay, and signals were detected by a microplate reader. (**N**) Necrotic cell death was measured using PI staining, and signals were detected by FACS. (*P < 0.05, **P < 0.01, and NS: not statistically significant compared to the control or DOX).

We observed that DOX treatment increased PARP1 expression and promoted necrosis. Therefore, we hypothesized that PARP1 may be an important factor contributing to DOX-induced necrosis. Thus, we used a pharmacological PARP1 inhibitor (PJ-34) to investigate whether PARP1 mediated the progression of DOX-induced necrosis, including DNA damage, mitochondrial hyper-activation, cATP production, oxidative stress, and cell death at 72 h after DOX stimulation. Our results suggested that PARP1 inhibition decreased the expression of PARP1, γ-H2AX, p-ATM, p-CHK2, and 53BP1, as compared to uninhibited, DOX-treated cells (Fig. 4A). Upon quantitative analysis, we found that the fluorescence intensities of p-ATM (25.0%) and γ-H2AX (41.3%) decreased in the nucleus in response to PARP1 inhibition, using a Cellomics ArrayScan HCS Reader, in at least 200 cells (Fig. 4B and Supplementary Information Figure S4). We further investigated the effect of PARP1 inhibition on necrosis and apoptosis using Annexin V and PI double staining. PARP1 inhibition significantly decreased the percentage of necrotic cells (47.1%), but did not significantly alter the percentage of apoptotic cells compared to DOX treatment alone (Fig. 4C). Furthermore, the inhibition of PARP1 reduced mitochondrial respiration (18.9%) and mitochondrial DNA (mtDNA) copy numbers, including those for NADH dehydrogenase subunit 1 (*ND1*; 34.3%) and NADH dehydrogenase subunit 4 (*ND4*; 33.9%) as shown in Fig. 4D,E. Inhibition of PARP1 suppressed total ATP (tATP; 48.8%) and cATP (40.6%) production levels compared to the levels in DOX-treated cells (Fig. 4F and Supplementary Information Figure S5D). DOX-induced cROS generation was also decreased (27.0%) after inhibition of PARP1, while mROS, cNO, and sNO production levels were not affected (Fig. 4G–J). The above-described pharmacological inhibition of PARP1 demonstrated that PARP1 increased mitochondrial hyper-activation, cATP production, and cROS generation, leading to necrosis in cells. Therefore, we further confirmed these findings by using siRNA-mediated *PARP1* knockdown in HK-2 cells at 72 h. PARP1 and γ-H2AX expression levels decreased at increasing concentration of *PARP1*-siRNA, and we chose 5 nM as the optimal concentration for subsequent experiments (Supplementary Information Figure S5A). *PARP1* knockdown suppressed the p-ATM expression, mtDNA (*ND4*) copy number (23.2%), cATP production (33.1%), and cROS generation (30.7%) as compared to that in DOX-treated cells transfected with scrambled siRNA (SC-siRNA; Fig. 4K,L and Supplementary Information Figure S5B and C). *PARP1* knockdown increased cell viability (29.0%) and decreased the frequency of PI-positive cells (23.6%) as compared to cells transfected with SC-siRNA and treated with DOX (Fig. 4M,N).

Necrosis, as evidenced by the presence of morphological changes, including loss of plasma membrane integrity and plasma membrane ruptures, which affected the permeability of the cells to PI dye, was observed. Therefore, we hypothesized that DOX-induced changes in the topography of the plasma membrane were suppressed by PARP1 inhibition and opted to use CNT AFM probes to measure these changes directly. CNT AFM probes have been shown to have the potential to acquire high-resolution images of biological materials, such as DNA and cells, due to the small tube diameter (ca. 3 nm), high aspect ratio, and chemical stability of CNTs^43^. This study is the first to provide direct evidence that HK-2 cells and PJ-34-treated cells exhibited smooth plasma membrane topography when viewed on 2–45-μm scale (Supplementary Information Figure S6A). Importantly, DOX-treated cells appeared to overflow (caused by cell swelling) when observed on the 45-μm scale and had obvious plasma membrane ruptures when viewed on 2–45-μm scales. Ruptures had an average depth of 162.6 ± 51.1 μm according to the line profile analysis. Inhibition of PARP1 prevented DOX-induced plasma membrane ruptures and also significantly reduced the rupture depth (51.7%) compared to DOX treatment alone (Supplementary Information Figure S6B). These findings suggested that DOX-induced mitochondrial hyper-activation, cATP production, cROS generation, and necrosis, but not mROS and NO generation, are mediated by PARP1.

### ROS contributes to DOX-induced DNA damage, cROS generation, cATP production, mitochondrial hyper-activation, and necrosis

**Figure 5:**
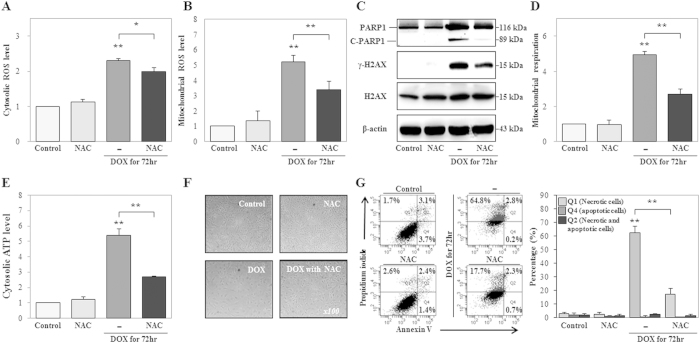
Treatment with a ROS scavenger suppresses doxorubicin (DOX)-induced cytosolic adenosine triphosphate (cATP) production, DNA damage, mitochondrial hyper-activation, and necrosis. HK-2 cells were treated with a ROS scavenger (N-acetyl cysteine, NAC, 5 mM), DOX (1 μM), or both DOX and NAC for 72 h. (**A**,**B**) Cytosolic ROS and mitochondrial ROS levels were measured using DCF-DA and MitoSOX, staining, respectively, and signals were detected by FACS analysis. (**C**) Expression levels of PARP1 (116 kDa), C-PARP1 (89 kDa), γ-H2AX^ser139^ (15 kDa), and H2AX were measured by western blot analysis in whole cell extracts. β-Actin was used as a loading control. The gels were electrophoresed under the same experimental conditions. (**D**,**E**) Mitochondrial respiration and cATP production were measured using MitoTracker Red CMXRos staining and ATP-based CellTiter-Glo Luminescent Cell Viability Assays, and signals were detected using FACS analysis or analysis on a microplate reader, respectively. (**F**) Morphological changes were measured using phase-contrast microscopy (original magnification, 100×). (**G**) Analysis of cell death, including necrosis (Q1), apoptosis (Q4), and necrosis with apoptosis (Q2), as measured using Annexin V and PI double staining. Necrotic and/or apoptotic cells were detected using FACS analysis. The histogram shows the percentage of cells in each group, as the average of the results of three independent experiments (*P < 0.05, **P < 0.01 compared to the control or DOX).

Previous studies reported that the generation of ROS contributes to DNA damage-induced necrosis or necroptosis via various intracellular signaling pathways^44^,^45^. Our results showed that DOX induced cROS and mROS generation, while PARP1 induced cROS generation. Thus, we next examined the influence of ROS on DOX-induced DNA damage, mitochondrial hyper-activation, cATP production, necrosis, and necrotic morphological changes by using the ROS scavenger, *N*-acetyl cysteine (NAC) in HK-2 cells at 72 h after treatment. In cells treated with both DOX and NAC, the levels of cROS (13.7%), mROS (34.9%), γ-H2AX, and C-PARP1 (89-kDa) decreased, while PARP1 levels were not affected (Fig. 5A,C). NAC decreased DOX-induced mitochondrial respiration (45.3%) and cATP production (50.1%) (Fig. 5D,E). Eventually, NAC protected cells from DOX-induced necrotic cell death (69.0%) and necrotic morphological changes of cell swelling compared to DOX (Fig. 5F,G). Thus, ROS generation contributed to DNA damage and mitochondrial hyper-activation, and enhanced DOX-induced necrotic cell death.

### ATP triggers DNA damage, cNO production, and cell death but does not induce cROS and mROS generation

**Figure 6:**
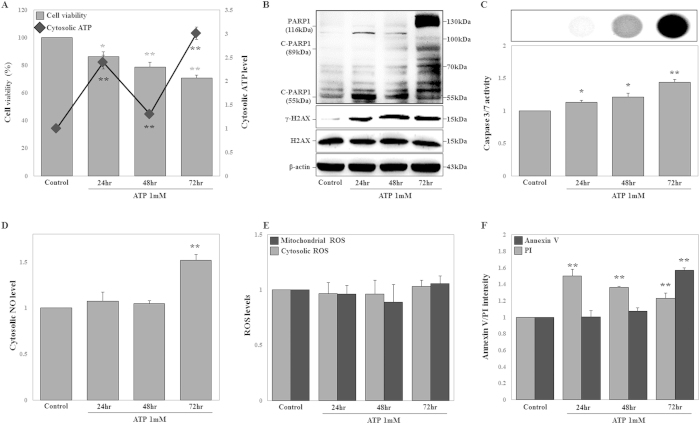
Adenosine triphosphate (ATP) treatment induces DNA damage and necrosis at the early stage and apoptosis at the late stage, but does not alter cytosolic reactive oxygen species (cROS) and mitochondrial reactive oxygen species (mROS) levels. HK-2 cells were treated with ATP (1 mM) for 24, 48, and 72 h. (**A**) Cell viability was measured using MTT assays. HK-2 and ATP-treated cells were counted using a hemocytometer and the same number of cells was seeded on plates. Cytosolic ATP was measured using ATP-based CellTiter-Glo Luminescent Cell Viability Assays. Signals were detected using a microplate reader. (**B**) Expression levels of PARP1 (116 kDa), C-PARP1 (89 kDa and 55 kDa), γ-H2AX^ser139^ (15 kDa), and H2AX (15 kDa) proteins were measured by western blot analysis in whole cell extracts. β-Actin was used as a loading control. (**C**) HK-2 and ATP-treated cells were counted using a hemocytometer and the same number of cells was seeded on plates. Caspase 3/7 activity was measured by Caspase-Glo 3/7 Assays, and signals were detected using a microplate reader, which measured luminescence intensity. Representative images are shown. (**D**,**E**) cROS, mROS, and cytosolic NO levels were measured using DCF-DA, MitoSOX, and DAF-FM staining, respectively, and signals were detected by FACS analysis. (**F**) Cell death, including necrosis (PI-positive cells) and apoptosis (Annexin V-positive cells), was measured using Annexin V or PI staining. Signals were detected by FACS analysis. The histogram shows the percentage of PI- and Annexin V-positive cells, as the average of the results of three independent experiments (*P < 0.05, **P < 0.01 compared to the control).

Our results suggested that the inhibition of PARP1 suppressed mitochondrial hyper-activation and cATP production levels during DOX-induced necrosis. Recent studies indicated that extracellular ATP is internalized by micropinocytosis in cancer cells, including human lung adenocarcinoma epithelial (A549) cells, colon carcinoma (RKO) cells, and breast adenocarcinoma (MCF7) cells^36^. Moreover, extracellular ATP triggers cell activities characteristic of apoptosis and necrosis, such as DNA fragmentation, apoptotic morphological changes, and cell swelling with loss of endoplasmic reticulum (ER) integrity^46^,^47^. Accordingly, we examined whether cATP regulated DNA damage, oxidative stress, and cell death in HK-2 cells at 24, 48, and 72 h after treatment. Preferentially, ATP (1 mM)-treated cells were harvested and the extracellular ATP removed, and the same number of cells were seeded in a plate for normalization of cell numbers. The cATP levels were measured using an ATP-based CellTiter-Glo Luminescent Cell Viability Assay. ATP internalization into the cells was noted at 24 h and 72 h and cell viability decreased in a time-dependent manner (Fig. 6A). Similarly, extracellular ATP was internalized into the cells, increasing tATP levels at 24 h (Supplementary Information Figure S7). Under these conditions, ATP-treated cells exhibited time-dependent increases in γ-H2AX protein expression, while C-PARP1 (55-kDa) increased only at 24 h, and PARP1 and C-PARP1 (89-kDa) increased only at 72 h (Fig. 6B). The number of PI-positive cells increased at 24 h and gradually decreased following ATP treatment, while caspase 3/7 activity, cNO production, and Annexin V-positive cells increased at 72 h (Fig. 6C,D,F). However, cROS and mROS generation was not significantly affected by ATP treatment (Fig. 6E). Thus, production of cATP contributed to DNA damage, cNO production, and cell death.

## Discussion

**Figure 7:**
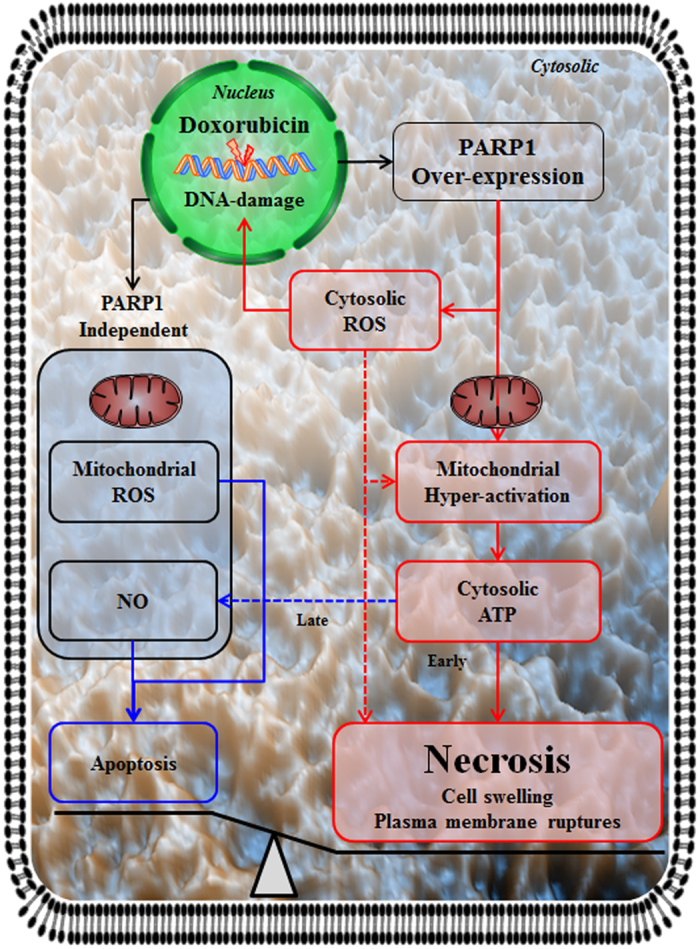
Overview of the mechanisms underlying PARP1-mediated necrosis by DNA damage. Doxorubicin-induced DNA damage causes PARP1 overexpression, promoting mitochondrial hyper-activation and inducing generation of cytosolic ATP. PARP1-dependent increases in cytosolic reactive oxygen species (ROS) enhance DNA damage, mitochondrial hyper-activation, and necrosis, while mitochondrial ROS and cytosolic nitric oxide (NO) production occurs independently of PARP1, and contribute to apoptosis. The generation of cytosolic ATP contributes to necrosis at the early stage and to apoptosis at the late stage. These events result in necrosis and morphological changes, such as cell swelling and plasma membrane rupture.

PARP1 is a nuclear protein, which by utilizing NAD^+^ and ATP is involved in single- and double-strand DNA break recognition, DNA damage repair, chromatin modification, and transcriptional regulation by poly-(ADP-ribosyl)ation^17^,^48^. Previous studies showed that PARP1 recognized double-strand DNA breaks and mediated the accumulation of mitotic recombination 11 (MRE11) and Nijmegen breakage syndrome 1 (NBS1) in double-strand DNA breaks induced by the DDR signaling pathway^49^,^50^. DNA strand breaks activate PARP1 and ATM (by means of ATM phosphorylation). PARP1-mediated PAR accumulation at sites of DNA damage contributes to the deployment of ATM at DNA-damaged sites and facilitates phosphorylation of its downstream targets, such as p53 and H2AX^51^. Although the mechanisms mediating necrosis or necroptosis have been intensively studied in recent years, the mechanisms through which PARP1 mediates necrosis and apoptosis and causes DNA damage are still not fully understood. Here, we elucidated the mechanisms by which PARP1 mediated necrosis, and show that it involves induction of mitochondrial hyper-activation, and increases in cATP production and cROS generation after DOX-induced DNA damage, in a p53-independent manner (Fig. 7).

Mitochondria are dynamic organelles that play an important bioenergetic role through F_1_F_0_-ATPase, using a proton electrochemical potential gradient in the intermembrane space that regulates both cellular metabolism and cell death signaling pathways in mammalian cells^52^,^53^,^54^. Transcription factors (transcription factor A, mitochondrial [TFAM]), co-activators (proliferator-activated receptor-gamma coactivator-1 α and β [PGC-1α and β], and nuclear respiratory factors 1 [NRF1]) promote mitochondrial biogenesis and regulate the antioxidant response as well as ATP production, which is closely associated with cell death^55^,^56^,^57^. A previous study had shown that ETO enhanced mitochondrial biogenesis as evidenced by increased expression of TFAM, PGC-1α, NRF1, and mtDNA copy numbers, and increased staining with mitochondrial targeting dyes through ATM-dependent activation of AMP-activated protein kinase (AMPK) in human adenocarcinoma (HeLa) cells^33^. In human T lymphocyte (Jurkat) and human promyelocytic leukemia (HL-60) cells, cytosolic mitochondrial oxygen consumption and cATP production levels increased during DOX-induced apoptotic cell death^58^. Eriocitrin also induced mitochondrial biogenesis as evidenced by increased *TFAM* and *NRF1* expression, staining with mitochondrial targeting dye, and mtDNA copy numbers. These changes resulted in cATP production in liver hepatocellular carcinoma (HepG2) cells and zebrafish^59^. It has also been reported that apoptotic stimulators, such as staurosporine, TNF-α, and ETO increased cATP production levels in HeLa, rat pheochromocytoma (PC12), and human histiocytic lymphoma (U937) cells. However, at middle and late stages, cATP levels and cell viability decreased, while caspase 3 activity and DNA fragmentation increased^60^. Moreover, activation of RIP3 induced mitochondrial bioenergetics by increasing the activities of metabolic enzymes involved in glycogenolysis, glycolysis, and glutaminolysis, and thus enhanced TNF-induced necrosis by inducing ROS generation in the mitochondria in response to hyper-activation of the respiratory chain^61^. A number of studies have shown that activation of PARP1 caused ATP depletion via the consumption of NAD^+^ and enhanced DNA damage-induced necrotic cell death^16^,^62^,^63^,^64^. Depletion of ATP through increased NAD^+^-consummation by PARP1 activation induced AMPK activation, caused PGC-1α-mediated TFAM activation, and initiated mitochondrial biogenesis. Furthermore, other transcription factors, such as NRF1 and peroxisome proliferator-activated receptors (PPARs), also contributed to mitochondrial biogenesis-related gene expression, including those related to the TCA cycle, mtDNA translation, and *TFAM*^65^. Recently, it was shown that ROS-induced DNA damage activated PARP1 and led to cATP depletion and AMPK activation in starved mouse embryonic fibroblasts (MEF)^66^. Activation of PARP1 caused ATP depletion and led to AMPK activation in DNA alkylating agents-induced necrosis^67^. Moreover, direct PARP1 interaction with NRF1 is involved in mitochondrial biogenesis^68^. Furthermore, DOX promoted the TCA cycle and mitochondrial respiration, and decreased cATP production levels in HL-1 cardiomyocytes. It is assumed that the enhancement of the TCA cycle and mitochondrial respiration occurs in order to compensate for the reduced cATP production levels^69^. Additionally, activation of PARP1 initiated ATP depletion in the mitochondria, while depletion of NAD^+^ did not affect cATP levels^70^. However, the molecular mechanism underlying PARP1-mediated mitochondrial dynamics and ATP production during cell death, including necrosis and apoptosis, is not fully understood. In this study, we demonstrated that ETO induced increases in mitochondrial mass, respiration, and ΔΨm, concurrent with increased C-PARP1 (89 kDa) expression, caspase 3 activity, and a loss of cell adhesion during necrotic and apoptotic cell death. In contrast, DOX increased mitochondrial respiration, ΔΨm, and mtDNA copy numbers, but did not cause changes in mitochondrial mass, corresponding to necrotic cell death-related morphological changes, such as nuclear and cell swelling, and plasma membrane rupture. The cATP production levels in both DOX- and ETO-treated cells increased compared to the cATP production levels in the controls. In DOX-treated cells, cATP production was higher than in ETO-treated cells. The inhibition of PARP1 by a pharmacological inhibitor or by siRNA reduced the DOX-induced increase in mtDNA copy number, mitochondrial respiration, cATP production, and necrotic cell death, suggesting that PARP1 may contribute to mitochondrial hyper-activation, cATP production, and necrosis. Interestingly, extracellular ATP was internalized, contributing to necrotic and apoptotic cell death. Taken together, these data supported the hypothesis that mitochondrial biogenesis occurs in necrotic and apoptotic cell death, while mitochondrial hyper-activation induces cATP production only in necrotic cell death. Mitochondria were hyper-activated in order to compensate for the PARP1-induced ATP depletion. Hyper-activation of mitochondria facilitated cATP production. It is most likely that the high ATP levels disrupt homeostasis and contribute to cell death. Additionally, the methods for measuring mitochondrial dynamics include the use of single- or double-staining with MitoTracker and JC-1, enabling the analysis of mitochondrial biogenesis and mitochondrial hyper-activation, and facilitating an understanding of cell death mechanisms.

Previous studies have shown that MOMP is initiated by expression of B-cell lymphoma protein 2 (BCL-2) homologous antagonist/killer (BAK) and BCL-2-associated X protein (BAX), and is prevented by expression of BCL2 during apoptosis^71^. MOMP normally induces the release of several cell death factors from the mitochondrial intermembrane space, including factors related to apoptotic cell death, such as cytochrome c, or non-apoptotic cell death, such as endonuclease G and AIF. AIF translocates into the nucleus and contributes to necrosis and apoptosis^35^,^36^. Our results showed that ETO-treatment of cells resulted in increased mature AIF in the nucleus, while DOX did not affect the translocation of mature AIF. Similarly, the expression of BCL2, a mitochondrial outer membrane protein, was not affected by DOX at 72 h, but ETO clearly induced BCL2 degradation. Taken together, DOX-induced necrosis was independent of AIF in our model.

Oxidative stress, including exposure to ROS and reactive nitrogen species (RNS), can be generated by different stressors that are closely associated with necrotic and apoptotic cell death signaling pathways, as well as cancer and other diseases^72^,^73^. ROS are commonly generated by respiratory chain complexes in the mitochondrial inner membrane, by NADPH oxidase in the plasma membrane, and in response to ER stress, and contribute to necrosis^74^,^75^. DOX can also induce the production of oxygen-derived free radicals, resulting in the generation of high levels of ROS^76^. However, the relationship between oxidative stress and apoptosis/necrosis has not been fully understood. Our results suggested that DOX-induced necrosis elevated oxidative stress and that PARP1 increases cROS generation in cells undergoing necrosis; in contrast, no changes in mROS or NO levels were observed. Moreover, DOX-induced DNA damage, mitochondrial hyper-activation, and ATP production increased, while necrosis was suppressed in cells treated with the ROS scavenger NAC. ATP-treated cells increased cNO production and, at later time points, increased C-PARP1 (89 kDa) expression, caspase 3 activity, and apoptosis. Thus, we believe that, following DOX-induced DNA damage, PARP1 initiates cROS generation independently of the mitochondria and causes mitochondrial hyper-activation/ATP production, eventually enhancing necrosis. On the other hand, mROS and NO production appear to be independent of PARP1 but contributed to mitochondria-dependent apoptosis through various apoptosis signaling factors.

p53, a tumor-suppressor protein, is an important transcription factor in antiapoptotic pathways and plays a critical role in cell cycle arrest. p53 also acts as an antioxidant for mild and repairable damage, while in cases of severe and irreparable damage, it promotes apoptosis^41^,^77^. p53 transcription increases the expression of numerous pro-apoptotic molecules, including BAX and p53-upregulated modulator of apoptosis (PUMA) which directly and indirectly leads to MOMP, and eventually activates cytochrome c-mediated apoptosis signaling pathways. Previous studies have shown that cisplatin induces apoptosis in a p53-dependent manner; however, the cell cycle arrest was independent of p53 in mouse testicular teratocarcinoma cells^78^. DOX-induced apoptosis proceeds independently of p53 in normal cells, including bovine aortic endothelial cells, but is dependent of p53 in tumor cells, including human ovarian teratocarcinoma cells^42^. Our results showed that DOX-induced expression of p53 did not affect DNA damage, cell cycle arrest, oxidative stress, or necrosis, indicating that the necrotic pathway was p53-independent. Thus, DOX-induced necrosis is mediated via necrotic signaling pathways independent of p53-mediated apoptotic signaling pathways. Furthermore, it is most likely that DOX will be more effective in the treatment of chemoresistant cancer, since mutated and dysfunctional p53 is one of the major causes of chemoresistance and occurs in more than 50% of cancers^79^.

PARP1 inhibitors, which can be used alone or in combination with DNA damage-inducing chemotherapy and radiotherapy, are effective against cancers with dysfunctional DNA repair^80^. In preclinical studies, PARP1 inhibitors have been reported to have therapeutic potential as treatment for cardiovascular diseases and Alzheimer’s disease, and as chemotherapeutic agents that may prevent unwanted necrosis^23^,^80^. In our study, we provided evidence that PARP1 plays a key role in regulating necrosis through mitochondrial hyper-activation and mediation of ATP production. Based on these results, we suggest that drugs targeting PARP1, mitochondrial respiration, and ATP synthase may hold potential as chemotherapeutic agents when combined with DNA damage-inducing drugs in cardiovascular diseases, Alzheimer’s disease, and cancer. Additionally, the methods used in this study may improve the analysis of mitochondrial dynamics and cell death.

## Materials and Methods

### Cell culture and treatments

Normal human kidney (HK-2) cells were purchased from the American Type Culture Collection (ATCC, Manassas, VA, USA). Cells were grown in RPMI1640 media (containing 1% penicillin/streptomycin and 10% fetal bovine serum; Thermo Fisher Scientific Inc., Waltham, MA, USA) and were incubated at 37 °C in an incubator (Thermo Fisher Scientific Inc.) in an atmosphere containing 5% CO_2_. DOX, ETO, ATP, and NAC were purchased from Sigma−Aldrich (St. Louis, MO, USA), and PJ-34 was purchased from Santa Cruz Biotechnology (Santa Cruz Biotechnology, Inc., Dallas, TX, USA).

### MTT assay for analysis of cell viability

Cells (5 × 10^3^/well) were seeded in 96-well plates (BD Biosciences., San Diego, CA, USA) and treated as indicated. 1-(4,5-Dimethylthiazol-2-yl)-3,5-diphenylformazan (MTT, Sigma−Aldrich) solution was added to the cells (100 μL/well), and cells were incubated at 37 °C for 3 h. MTT solution was then removed, and DMSO was added (100 μL/well). After a 30 min incubation, the absorbance was detected using a microplate spectrophotometer (Molecular Devices Inc., Sunnyvale, CA, USA) at 540 nm.

### Cytosolic ATP analysis

Cells (4 × 10^5^ per 6-cm dish) were plated (SPL Life Sciences, Pochun, Korea) and treated as indicated. Cells were harvested by centrifugation at 200 × *g* for 3 min to remove extracellular ATP. Harvested cells were counted using a hemocytometer (Paul Marienfeld GmbH & Co., KG, Bad Mergentheim, Germany) and seeded at a density of 1 × 10^4^ cells per well in 96-well plates (Greiner Bio-One, Frickenhausen, Germany). Next, 50 μL ATP-based CellTiter-Glo Luminescent Cell Viability Assay solution (Promega, Madison, WI, USA) was added, and cells were incubated for 10 min. The luminescence intensity was measured using a Molecular Devices SPECTRAmax GEMINI fluorescence microplate reader (Molecular Devices Inc.) and/or a Fuji LAS-3000 system (Fujifilm, Tokyo, Japan).

### Total ATP analysis

Cells (4 × 10^5^ per 6-cm dish) were plated (SPL Life Sciences) and treated as indicated. Cells were harvested by centrifugation at 200 × *g* for 3 min to remove the extracellular ATP. Harvested cells were counted using a hemocytometer (Paul Marienfeld GmbH & Co.). Total ATP levels from 1 × 10^5^ cells were measured using an ENLITEN^®^ ATP Assay System Bioluminescence Detection kit (Promega) according to the manufacturer’s protocol. The luminescence intensity was measured using a Molecular Devices SPECTRAmax GEMINI fluorescence microplate reader (Molecular Devices Inc.).

### NO secretion analysis

Cells (4 × 10^5^ per 6-cm dish) were plated (SPL Life Sciences) and treated as indicated. A syringe filter (pore size: 0.22 μm; BD Biosciences) was used to filter supernatant media (1 mL). One-hundred-microliter samples of supernatant media were added to the wells of 96-well plates (BD Biosciences). N2 (50 μL), and N1 buffers (50 μL; iNtRON Biotechnology, Seoul, Korea) were immediately added and the samples were incubated overnight at room temperature. The absorbance was detected using a microplate spectrophotometer reader (Molecular Devices Inc.) at 550 nm.

### Caspase 3/7 activity analysis

Cells (4 × 10^5^ per 6-cm dish) were plated (SPL Life Sciences) and treated as indicated. Cells were harvested by centrifuged at 200 × *g* for 3 min to remove debris. Harvested cells were counted using a hemocytometer (Paul Marienfeld GmbH & Co.) and seeded in 96-well plates (Greiner Bio-One) at a density of 1 × 10^4^ cells per well. Caspase 3/7 activity was measured using a Caspase-Glo 3/7 Assay kit (Promega) according to the manufacturer’s protocol. The luminescence intensity was measured using a Molecular Devices SPECTRAmax GEMINI fluorescence microplate reader (Molecular Devices Inc.) and/or a Fuji LAS-3000 system (Fujifilm).

### Confocal microscopy analysis

Cells (1 × 10^4^/well) were fixed with 3.7% formaldehyde (Sigma−Aldrich) for 15 min and permeabilized with 0.2% Triton X-100 for 15 min. Subsequently, cells were blocked with 5% fetal bovine serum in phosphate-buffered saline (PBS; AMRESCO, Solon, OH, USA) for 1 h and then incubated with primary antibodies (1:1000 dilution, 1 h) targeting γ-H2AX, PARP1, p-p53^ser15^, and p-ATM (Santa Cruz Biotechnology, Inc.). Cells were then incubated with secondary antibodies (1:1000 dilution, 1 h) conjugated with Alexa Fluor 488 or 546 (Invitrogen, Carlsbad, CA, USA). Nuclei were stained with Hoechst 33258 reagent (5 μM; Sigma−Aldrich) for 15 min. Stained cells were imaged using confocal microscopy (LSM-700, Carl Zeiss Microimaging, Oberkochen, Germany) and analyzed using Zen 2009 software.

### Phase contrast microscopy

Cells (4 × 10^5^) were seeded in 6-cm dishes and allowed to grow overnight in an incubator containing 5% CO_2_ at 37 °C. Media were replaced and cells were treated under the conditions indicated. Morphological changes were examined using phase-contrast microscopy (E-Scope i304, Macrotech Corp., Goyang, Korea) and photographed using Scopephoto software.

### Analysis of nuclear area using a Cellomics ArrayScan HCS Reader

Cells (5 × 10^3^/well) were seeded in a 96-well plate (Greiner Bio-One) and treated as indicated. Cells were fixed with 3.7% formaldehyde (Sigma−Aldrich) for 15 min and stained with Hoechst 33258 reagent (5 μM, Sigma−Aldrich). Stained cells were analyzed using a Cellomics ArrayScan HCS Reader (Thermo Fisher Scientific), and the nuclear area was calculated using ArrayScan VTI (600 series) Version 6.6.1.3 software, with at least 200 cells per sample.

### Analysis of nuclear protein expression using a Cellomics ArrayScan HCS Reader

Cells (5 × 10^3^/well) were seeded on clear-bottom black 96-well plates (Greiner Bio-One) and treated as indicated. Cells were fixed with 3.7% formaldehyde (Sigma−Aldrich) for 15 min and permeabilized with 0.2% Triton X-100 for 15 min. Cells were then blocked with 5% fetal bovine serum in PBS (AMRESCO) for 1 h and incubated with primary antibodies (1:1000 dilution, 1 h) targeting γ-H2AX, PARP1, and p-ATM (Santa Cruz Biotechnology, Inc.). After incubation with primary antibodies, cells were incubated with secondary antibodies (1:1000 dilution, 1 h) conjugated with Alexa Fluor 488 or 546 (Invitrogen). Nuclei were stained with Hoechst 33258 reagent (5 μM) for 15 min. Stained cells were measured using a Cellomics ArrayScan HCS Reader (Thermo Fischer Scientific) and fluorescence intensities were analyzed using ArrayScan VTI (600 series) Version 6.6.1.3 software, with at least 200 cells per sample.

### Analysis of cell cycle distribution by PI staining

Cells (4 × 10^5^ per 6-cm dish) were plated (SPL Life Sciences) and treated as indicated. Harvested cells were washed with 1 mL PBS (AMRESCO) and centrifuged at 200 × *g* for 3 min. Next, ice-cold 70% ethanol in PBS (AMRESCO) was added, and cells were incubated at 4°C overnight. After centrifugation at 200 × *g* for 3 min, cells were washed with 1 mL PBS and labeled with PI (50 μg/mL; Sigma−Aldrich) and RNase A (500 μg/mL; Sigma−Aldrich) for 15 min. Stained cells were analyzed using a FACSAria III instrument with Diva software (BD Biosciences).

### Analysis of apoptosis and necrosis by Annexin V/PI staining

Cells (4 × 10^5^ per 6-cm dish) were plated (SPL Life Sciences) and treated as indicated. Harvested cells were washed with 1 mL PBS (AMRESCO) and centrifuged at 200 × *g* for 3 min. Next, 100 μL binding buffer was added in brown tubes, and cells were labeled with Annexin V (10 μL; BD Biosciences) and/or PI (10 μL; BD Biosciences) for 15 min. Two hundred microliters of binding buffer were then added, and samples were stored at 4 °C. Stained cells were analyzed using a FACSAria III instrument with Diva software (BD Biosciences).

### Analysis of mROS using MitoSOX staining

Cells (4 × 10^5^ per 6-cm dish) were plated (SPL Life Sciences) and treated as indicated. Harvested cells were washed with 1 mL PBS (AMRESCO) and centrifuged at 200 × *g* for 3 min. Cells were then incubated with MitoSOX (5 μM; Invitrogen) for 10 min at 37°C in an incubator with 5% CO_2_. Cells were collected by trypsinization for 2 min and centrifuged at 200 × *g* for 5 min. The supernatants were then removed, and 300 μL PBS (AMRESCO) was added to the pellets. Pellets were stored on ice until analysis. Stained cells were analyzed using a FACSAria III instrument with Diva software (BD Biosciences).

### Analysis of mitochondrial respiration and mitochondrial mass by MitoTracker CMXRos and green FM staining

Cells (4 × 10^5^ per 6-cm dish) were plated (SPL Life Sciences) and treated as indicated with carbonyl cyanide 3-chlorophenylhydrazone (50 μM; Invitrogen) for 5 min or DOX or ETO for 72 h. Cells were incubated with MitoTracker CMXRos and/or green FM (100 nM; Invitrogen) for 15 min at 37 °C in 5% CO_2_ and were then collected by trypsinization for 2 min. Harvested cells were centrifuged at 200 × *g* for 5 min. Supernatants were then removed, and 300 μL PBS (AMRESCO) was added to the cells. Samples were stored on ice until analysis using a FACSAria III instrument with Diva software (BD Biosciences).

### Analysis of mitochondrial mass and outer membrane potential by JC-1 staining

Cells (4 × 10^5^ per 6-cm dish) were plated (SPL Life Sciences) and treated as indicated with carbonyl cyanide 3-chlorophenylhydrazone (Invitrogen) for 5 min. Cells were incubated with JC-1 (2 μM; Invitrogen) for 15 min at 37 °C in an incubator with a 5% CO_2_ atmosphere. Cells were collected by trypsinization for 2 min and were centrifuged at 200 × *g* for 5 min. Supernatants were removed, and cells were stored in 300 μL PBS (AMRESCO) on ice until required for use. Stained cells were analyzed using a FACSAria III instrument with Diva software (BD Biosciences).

### Analysis of cNO and cROS by DAF-FM and DCF-DA staining

Cells (4 × 10^5^ per 6-cm dish) were plated (SPL Life Sciences) and treated as indicated. Harvested cells were washed with 1 mL PBS (AMRESCO) and centrifuged at 200 × *g* for 3 min. Cells were incubated with DAF-FM (5 μM; Invitrogen) or DCF-DA (10 μM; Invitrogen) at 37 °C for 1 h or 15 min, respectively. Following incubation, cells were centrifuged at 200 × *g* for 5 min in brown tubes; 300 μL PBS (AMRESCO) was added, and cells were stored on ice until required for use. Stained cells were analyzed using a FACSAria III instrument with Diva software (BD Biosciences).

### Analysis of mtDNA copy number by real-time PCR

Cells (4 × 10^5^ per 6-cm dish) were plated (SPL Life Sciences) and treated as indicated. Total genomic DNA was extracted using a QuickGene SP kit (Fujifilm) according to the manufacturer’s protocol. The genomic DNA concentration was measured using a Micro UV-Vis fluorescence spectrophotometer (Malcom, Tokyo, Japan). The mtDNA copy number was examined using a QuantiTect SYBR Green PCR kit (Qiagen, Valencia, CA, USA) and real-time PCR was performed on a Rotor-Gene Q system (Qiagen) with primers for mtDNA, including *ND1* and *ND4*, as described previously^81^ and for *GAPDH* (5′-CCA CCC ATG GCA AAT TCC ATG GCA-3′ and 5′-TCT AGA CGG CAG GTC AGG TCC ACC-3′). The two-step PCR protocol was as follows: 10 min at 95 °C, followed by 30 cycles of denaturing (5 s at 95 °C) and annealing (10 s at 57 °C). Relative mtDNA levels were calculated using the ΔΔCT method, and mtDNA levels were normalized to *GAPDH* levels using the Rotor-Gene Q Series software.

### Analysis of protein expression by western blotting

Whole protein extraction solution (M-PER; Thermo) containing protease and phosphatase inhibitor cocktails (Thermo) was added to cell pellets, and cells were incubated at 4 °C for 10 min. Cells were collected by centrifugation at 16,000 × *g* for 10 min. Protein concentrations were measured by the bicinchoninic acid assay (BCA kit; Sigma-Aldrich), and equal amounts of protein (50–60 μg) were separated on 10%–12% polyacrylamide gels using electrophoresis. Proteins were transferred to nitrocellulose membranes (Hybond ECL; Amersham Pharmacia Biotech Inc., Piscataway, NJ, USA) in transfer buffer. Transfer conditions were as follows: 70 V for 4 h at 4 °C. Membranes were blocked with 0.05% nonfat dried milk solution in deionized water for 1 h and were then incubated with primary antibodies (1:500–1:1000) targeting γ-H2AX, PARP1, MDM2, p-p53^ser15^, p-p53^ser392^, p-ATM, 53BP1, BRCA1, BCL2, and β-actin (Santa Cruz Biotechnology, Inc.) overnight at 4°C with gentle agitation. The H2AX, ATM, p53, CHK2, cleaved-caspase 3, and p-CHK2 antibodies obtained from Cell Signaling Technology (Danvers, MA, USA) were also used. The next day, membranes were incubated with peroxidase-conjugated anti-mouse or anti-rabbit antibodies (1:1000 dilution) for 2 h and washed with PBS (AMRESCO) containing 0.05% Tween-20 (PBST). Protein detection was facilitated by SuperSignal West Pico ECL solution (Thermo Fisher Scientific), and signals were visualized by developing X-ray films or by imaging on a Fuji LAS-3000 system (Fujifilm).

### Analysis of cell swelling using xCELLigence system

Cells (4 × 10^5^) were seeded in 6-cm dishes (SPL Life Sciences) and allowed to grow overnight at 37 °C in an incubator containing 5% CO_2_. The next day, the medium was changed, and cells were treated with DOX and ETO for 72 h. Cells were then harvested, counted using a hemocytometer (Paul Marienfeld GmbH & Co.), and 1 × 10^4^ cells were seeded on an E-plate device (ACEA Biosciences, Inc., San Diego, CA, USA). Real-time monitoring of electrical impedance (cell index) was measured using an xCELLigence system (Roche Diagnostics, Mannheim, Germany) at 10-s intervals for 2.5 h. Data were analyzed using RTCA DP software (version 1.2).

### Analysis of cell morphology and plasma membrane topography using CNT/AFM probes

Cells (1 × 10^4^ per 6-cm dish) were plated (SPL Life Sciences), pretreated with PJ-34 for 1 h, and subsequently co-treated with DOX for 72 h. Supernatants were removed, and cells were fixed with 3.7% formaldehyde for 15 min. Fixed cells were washed with PBS (AMRESCO) and deionized water and were then dried. All AFM images were acquired on an XE-100 AFM system (Park Systems Corp., Suwon, Korea) in noncontact AFM mode with a CNT/AFM cantilever, which had a resonance frequency of 310 kHz. AFM image analysis was carried out using XEI software (Park Systems, Inc.).

### Knockdown of *PARP1* using siRNA

Cells (2 × 10^5^ per 6-cm dish) were plated (SPL Life Sciences) and allowed to grow overnight at 37 °C in an incubator containing 5% CO_2_. The next day, cells were transfected with *PARP1* siRNA (Santa Cruz Biotechnology, Inc.) using FuGENE HD transfection reagent (Promega) according to the manufacturer’s protocol. After 24 h, the medium was changed, and samples were used for experiments.

### Statistical analysis

All histogram data are representative of experiments performed independently at least three times. Data were analyzed using one-way analysis of variance in SigmaPlot software (Systat Software Inc, San Jose, USA). Differences were considered significant when *P-*values were less than 0.05.

## Additional Information

**How to cite this article**: Shin, H.-J. *et al.* Doxorubicin-induced necrosis is mediated by poly-(ADP-ribose) polymerase 1 (PARP1) but is independent of p53. *Sci. Rep.* **5**, 15798; doi: 10.1038/srep15798 (2015).

## Supplementary Material

Supplementary Information
