# Supplementary material for: Doxorubicin-induced necrosis is mediated by poly-(ADP-ribose) polymerase 1 (PARP1) but is independent of p53
Source: Sci Rep. 2015 Nov 2;5:15798. doi: 10.1038/srep15798 (PMC4629133; doi:10.1038/srep15798)
Supplement: Supplementary Information [file srep15798-s1.pdf]

< **Supplementary Information** >

Doxorubicin-induced necrosis is mediated by poly-(ADP-ribose) polymerase 1 (PARP1) but is independent of p53

Hyeon-Jun Shin[1], Hyuk-Kwon Kwon[1], Jae-Hyeok Lee, Xiangai Gui, Asma Achek, Jae-Ho Kim & Sangdun Choi[*]

Department of Molecular Science and Technology, Ajou University, Suwon 443-749, Korea

[1]These authors contributed equally to this work

*Corresponding author:
Sangdun Choi
Department of Molecular Science and Technology, Ajou University, Suwon 443-749, Korea
Phone: +82-31-219-2600
Fax: +82-31-219-1615
E-mail: sangdunchoi@ajou.ac.kr

Running title:
DNA damage-induced necrosis mediated by PARP1

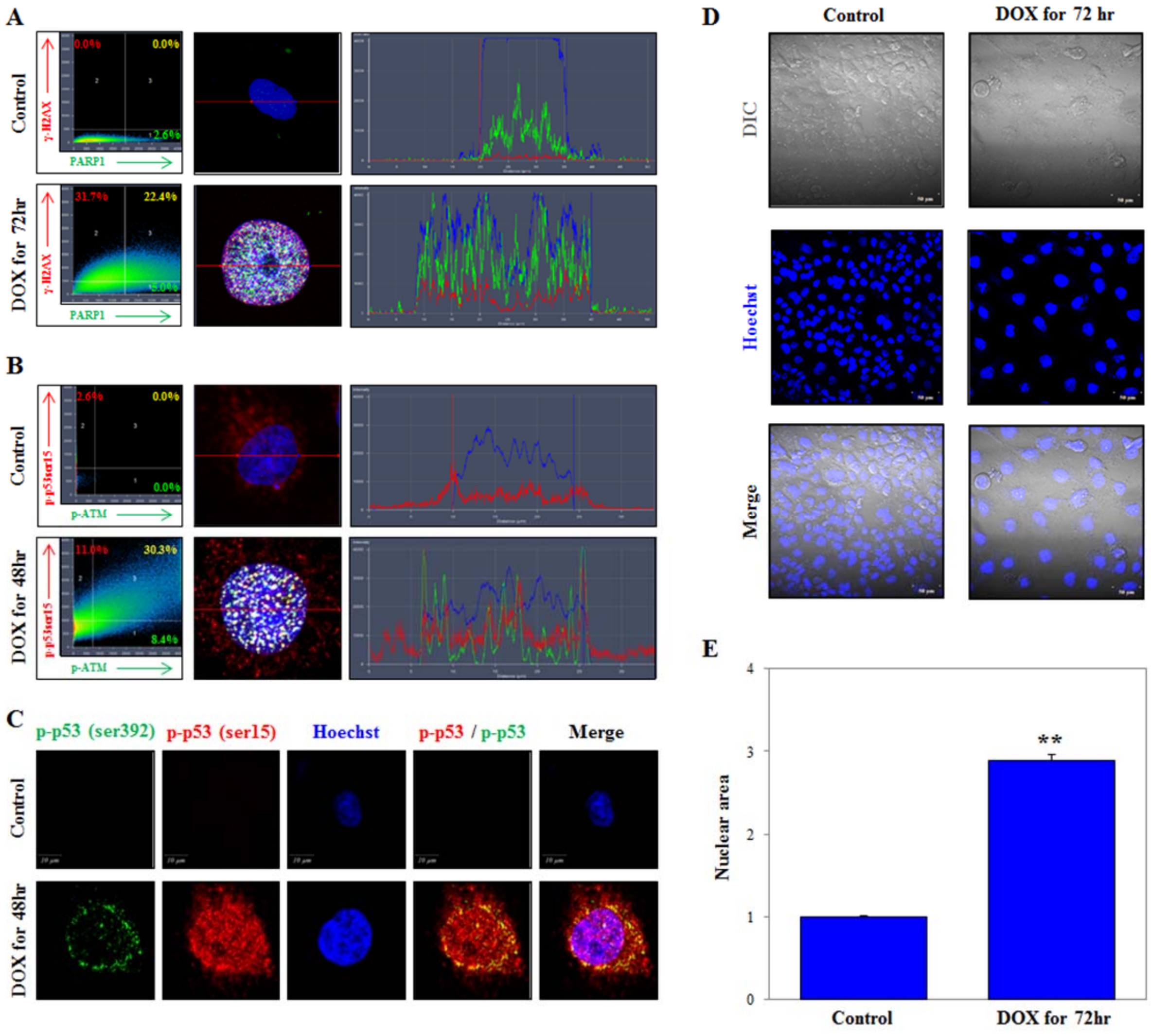

Figure S1 | Doxorubicin (DOX) increases the expression of γ-H2AX and PARP1 and the phosphorylation of ATM, p53^{ser15}, and p53^{ser392}, and induces nuclear swelling. HK-2 cells were treated with DOX (1 μM) for the indicated times. (A and B) Expression of γ-H2AX/PARP1 and p-p53^{ser15}/ATM were measured using immunofluorescence staining and confocal microscopy. The percent colocalization in the nucleus was analyzed using Zen 2009 software; region 1 (green fluorescence), region 2 (red fluorescence), and region 3 (green and red colocalization) are shown. Line profiles indicate average fluorescence intensities of the red line. (C) Levels of p-p53^{ser15}/p-p53^{ser392} were measured using immunofluorescence staining and confocal microscopy. Nuclei were stained with Hoechst (blue color). Scale bar: 10 μm. (D and E) Nuclear swelling was measured using Hoechst staining (blue color), and staining was detected by confocal microscopy (scale bar: 50 μm). The nuclear area was

quantified using a Cellomics ArrayScan HCS Reader with at least 200 cells per sample. Histograms shown are the average of the results of three independent experiments (* $P < 0.05$, ** $P < 0.01$ compared to the control).

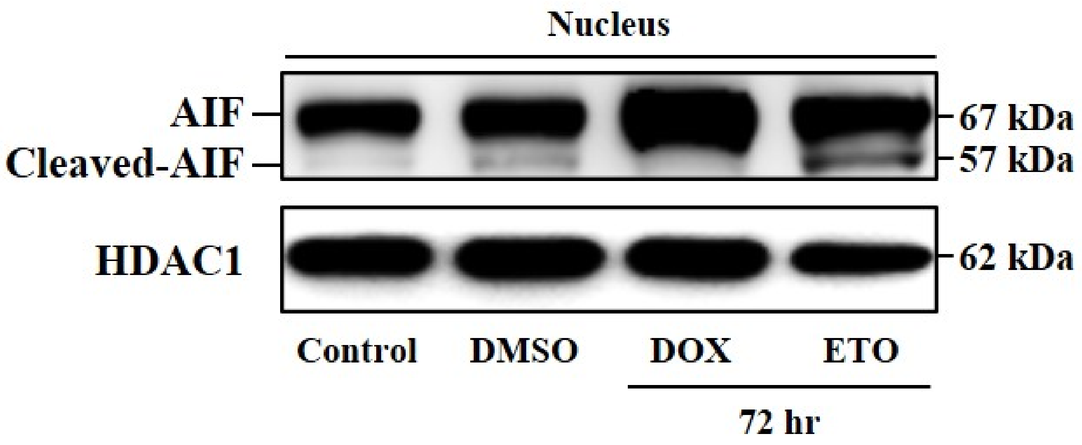

Figure S2 | Doxorubicin (DOX) and etoposide (ETO) differentially regulate AIF translocation. HK-2 cells were treated with DOX (1 μM) and ETO (50 μM) for 72 h. Expression levels of AIF were measured by western blot analysis in nuclear extracts. HDAC1 was used as a loading control.

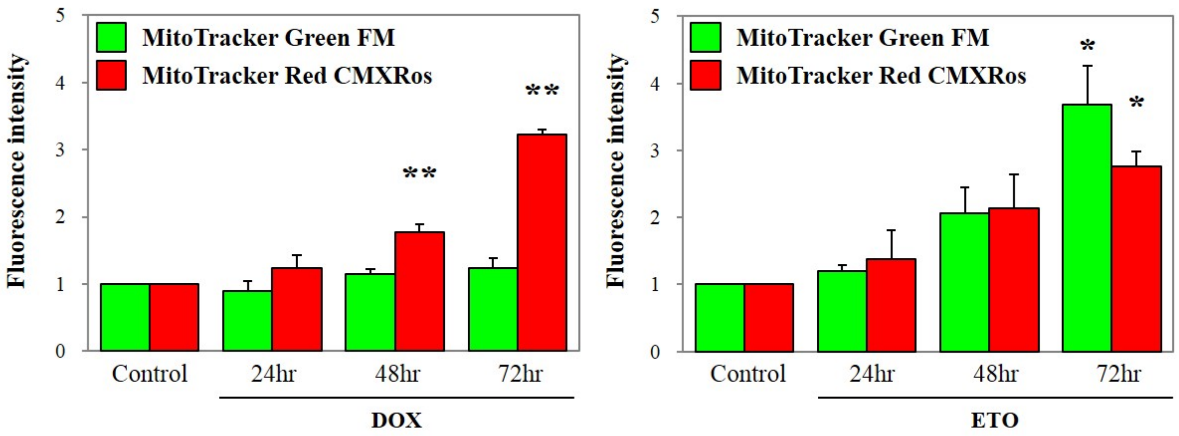

Figure S3 | Doxorubicin (DOX) and etoposide (ETO) differentially regulate mitochondrial mass and respiration. Mitochondrial mass and respiration were measured using MitoTracker Green FM and Red CMXRos, respectively. Detection was facilitated by FACS analysis. The histogram shows fluorescence intensities as the average of the results of three independent experiments (* $P < 0.05$, ** $P < 0.01$ compared to the control).

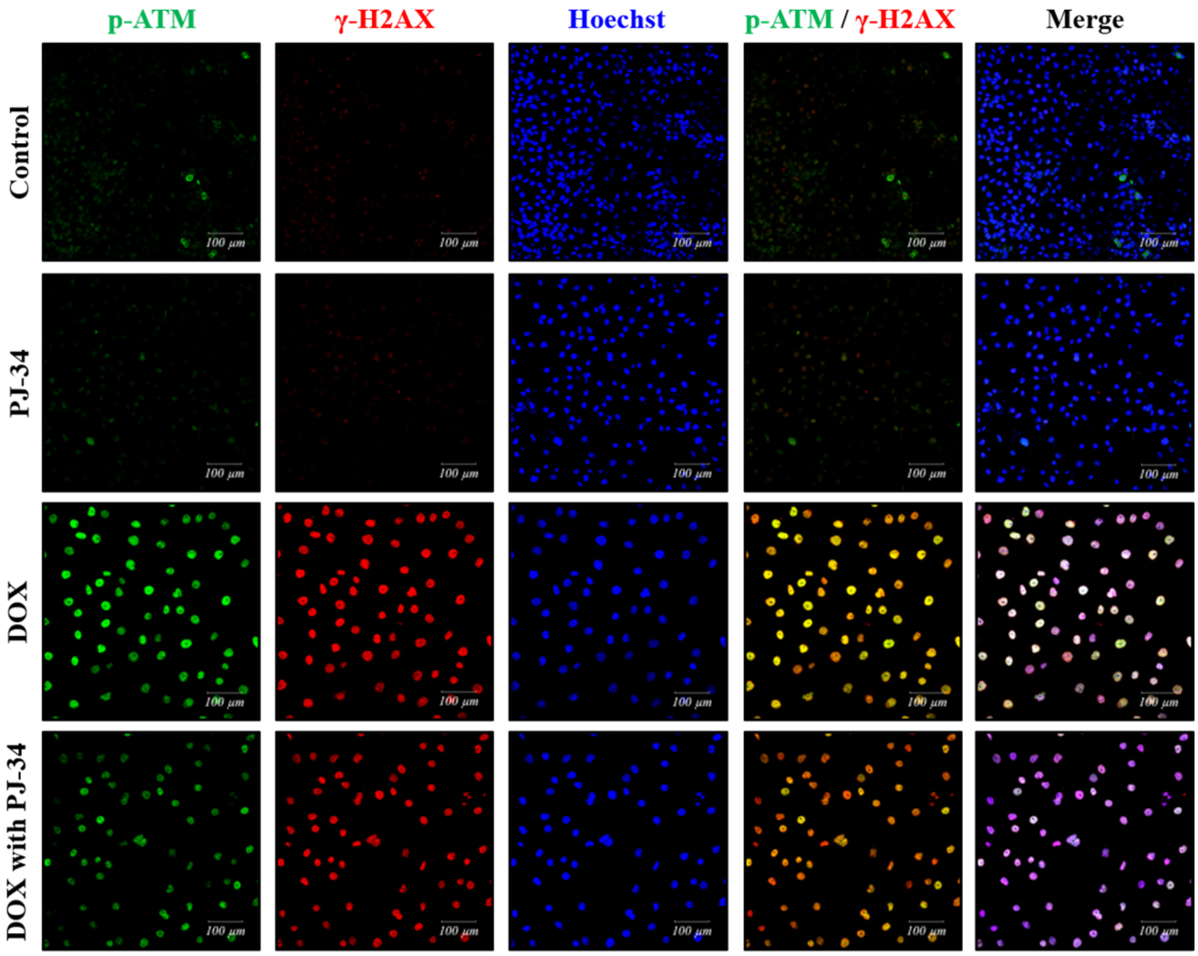

Figure S4 | Inhibition of PARP1 suppresses doxorubicin (DOX)-induced expression of γ-H2AX and p-ATM. HK-2 cells were treated with PJ-34 (10 μM), DOX (1 μM), or both DOX and PJ-34 for 72 h. p-ATM and γ-H2AX fluorescence intensities were determined using immunofluorescence staining and confocal microscopy. Nuclei were stained with Hoechst stain (blue color). Scale bar: 100 μm.

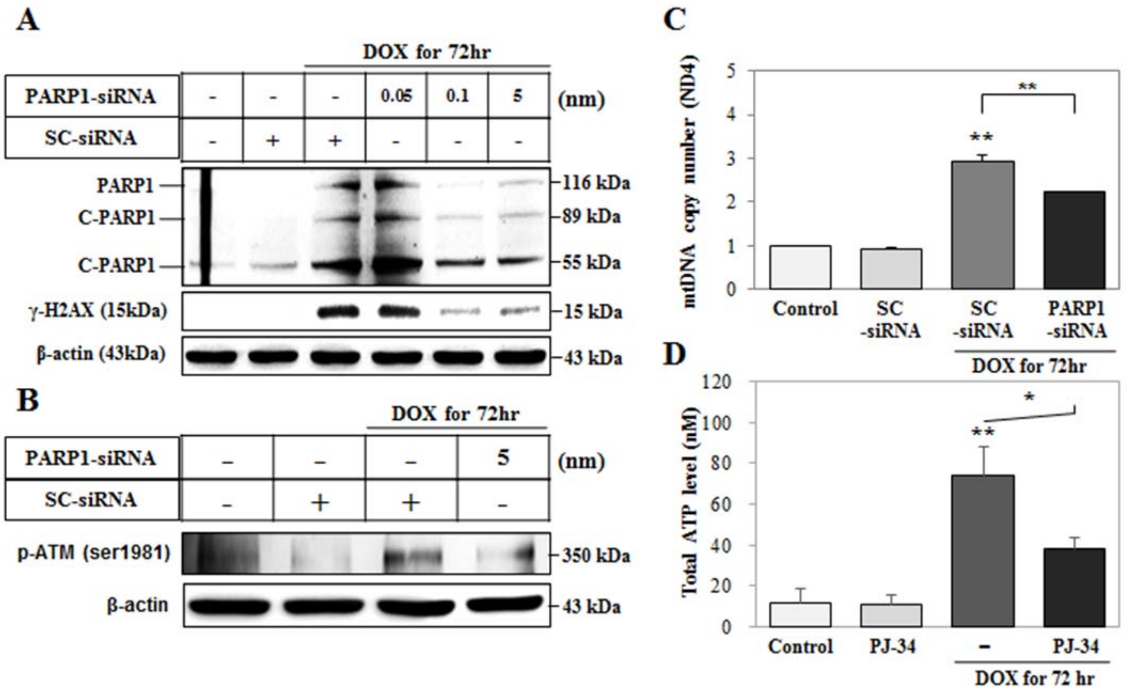

Figure S5 | Knockdown of *PARP1* suppresses doxorubicin (DOX)-induced DNA damage, p-ATM levels, and mitochondrial DNA copy number. (A) HK-2 cells were transfected with SC-siRNA (5 nM) or *PARP1*-siRNA (0.05, 0.1, or 5 nM) and treated with DOX for 72 h. Expression levels of PARP1 and γ-H2AX proteins were measured by western blot analysis in whole cell extracts. β-Actin was used as a loading control. (B) HK-2 cells were transfected with SC-siRNA (5 nM) or *PARP1*-siRNA (5 nM) and treated with DOX for 72 h. Levels of p-ATM were measured by western blot analysis in whole cell extracts. β-Actin was used as a loading control (C) Genomic DNA was analyzed for mtDNA copy number using *ND4* and *GAPDH* primers and real-time PCR. *ND4* copy numbers were normalized to *GAPDH* copy numbers, and normalized data were compared to data from control samples. (D) HK-2 cells were treated with a pharmacological PARP1 inhibitor (PJ-34, 10 μM), DOX (1 μM), or both PJ-34 and DOX for 72 h. HK-2 cells and treated cells were counted using a hemocytometer. Total ATP levels were measured using an ENLITEN® ATP Assay System Bioluminescence Detection kit and detected using a microplate reader. All histograms shown are the average of the results of three independent experiments (* $P < 0.05$, ** $P < 0.01$ compared to control or SC-siRNA with DOX).

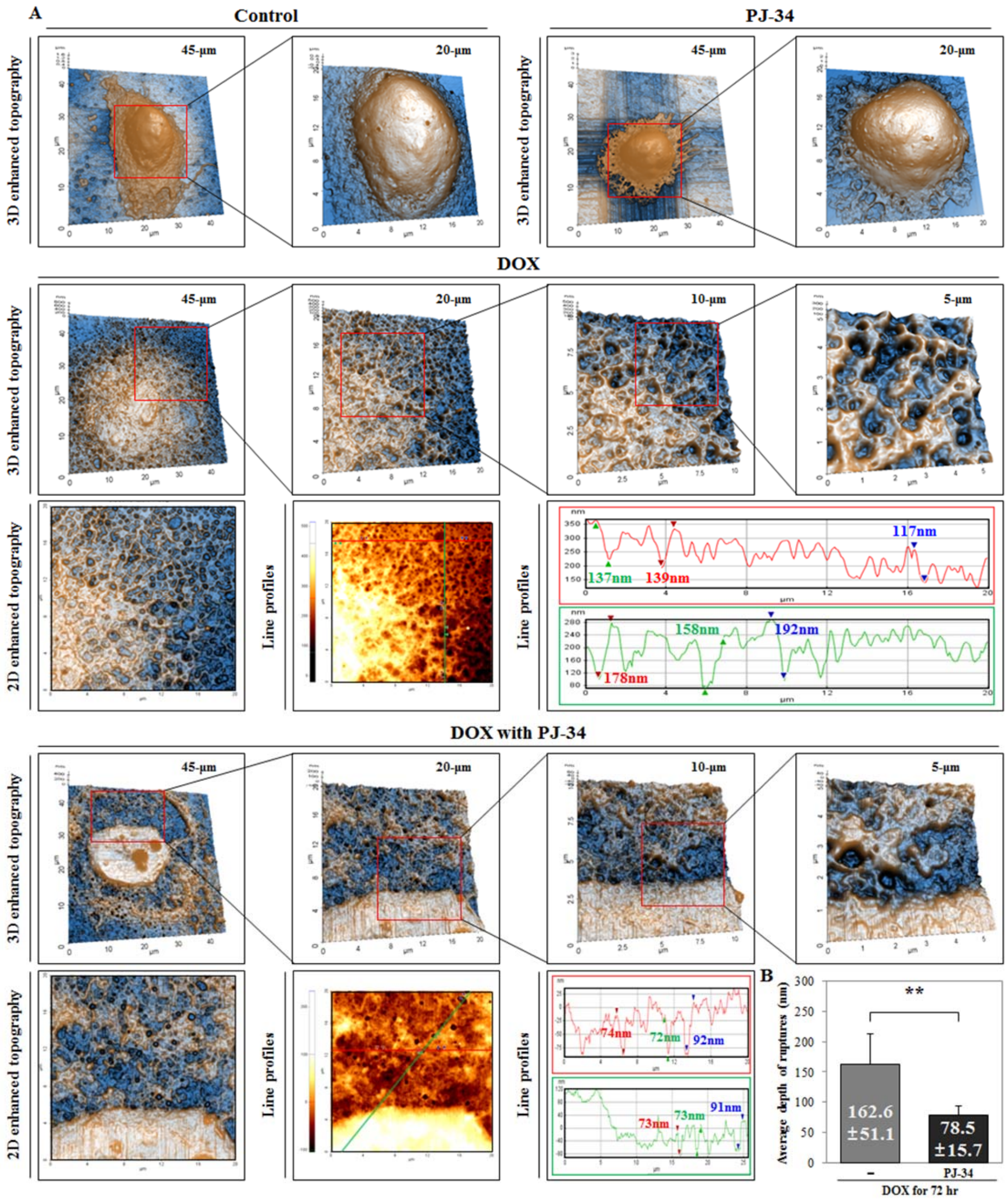

Figure S6 | Inhibition of PARP1 suppresses doxorubicin (DOX)-induced necrosis and plasma membrane rupture. HK-2 cells were treated with PJ-34 (10 µM), DOX, or both DOX and PJ-34 for 72 h. Plasma membrane topography changes were measured using CNT/AFM probe analysis from 2 to 45 µm. (A) Images show 3D-enhanced color topography at the indicated scales and were analyzed using XEI software. Plasma membrane ruptures and

roughness were measured using line profile analysis, including red and green lines in 2D topography (20-μm scale) for 10 cases. (B) The average rupture depth of each sample is shown in the histogram, and represents the average of the results of three independent experiments (* $P < 0.05$, ** $P < 0.01$ compared to DOX).

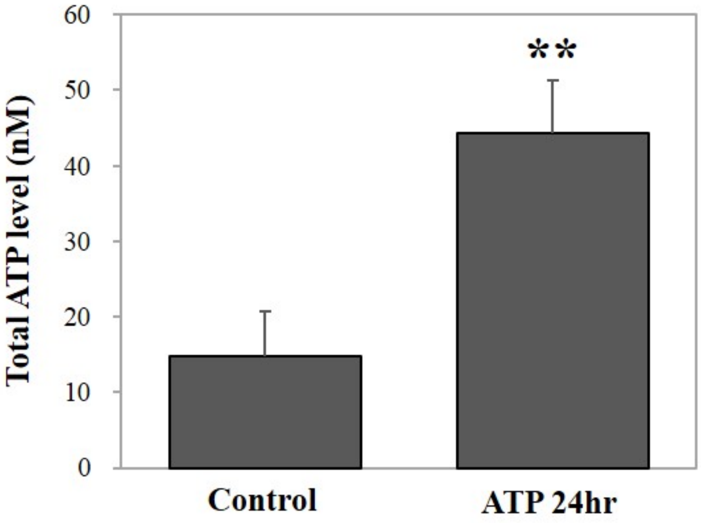

Figure S7 | Extracellular adenosine triphosphate (ATP) is internalized. HK-2 cells were treated with ATP (1 mM) for 24 h and treated cells were counted using a hemocytometer. Total ATP levels were measured using an ENLITEN® ATP Assay System Bioluminescence Detection kit and detected using a microplate reader. Histogram data represent the average of the results of three independent experiments (* $P < 0.05$, ** $P < 0.01$ compared to control).
